# The risk profile of patients with COVID-19 as predictors of lung lesions severity and mortality—Development and validation of a prediction model

**DOI:** 10.3389/fmicb.2022.893750

**Published:** 2022-07-25

**Authors:** Ezat Rahimi, Mina Shahisavandi, Albert Cid Royo, Mohammad Azizi, Said el Bouhaddani, Naseh Sigari, Miriam Sturkenboom, Fariba Ahmadizar

**Affiliations:** ^1^Clinical Research Unit, Department of Internal Medicine, Kowsar Hospital, Kurdistan University of Medical Sciences, Sanandaj, Iran; ^2^Epilepsy Research Center, Shiraz University of Medical Sciences, Shiraz, Iran; ^3^Department of Datascience and Biostatistics, University Medical Center Utrecht, Utrecht, Netherlands; ^4^School of Medicine, Kurdistan University of Medical Sciences, Sanandaj, Iran; ^5^Lung Diseases and Allergy Research Center, Research Institute for Health Development, Kurdistan University of Medical Sciences, Sanandaj, Iran

**Keywords:** COVID-19, coronavirus, machine learning, mortality, lung injury

## Abstract

**Objective:**

We developed and validated a prediction model based on individuals' risk profiles to predict the severity of lung involvement and death in patients hospitalized with coronavirus disease 2019 (COVID-19) infection.

**Methods:**

In this retrospective study, we studied hospitalized COVID-19 patients with data on chest CT scans performed during hospital stay (February 2020-April 2021) in a training dataset (TD) (*n* = 2,251) and an external validation dataset (eVD) (*n* = 993). We used the most relevant demographical, clinical, and laboratory variables (*n* = 25) as potential predictors of COVID-19-related outcomes. The primary and secondary endpoints were the severity of lung involvement quantified as mild (≤25%), moderate (26–50%), severe (>50%), and in-hospital death, respectively. We applied random forest (RF) classifier, a machine learning technique, and multivariable logistic regression analysis to study our objectives.

**Results:**

In the TD and the eVD, respectively, the mean [standard deviation (*SD*)] age was 57.9 (18.0) and 52.4 (17.6) years; patients with severe lung involvement [*n* (%):185 (8.2) and 116 (11.7)] were significantly older [mean (*SD*) age: 64.2 (16.9), and 56.2 (18.9)] than the other two groups (mild and moderate). The mortality rate was higher in patients with severe (64.9 and 38.8%) compared to moderate (5.5 and 12.4%) and mild (2.3 and 7.1%) lung involvement. The RF analysis showed age, C reactive protein (CRP) levels, and duration of hospitalizations as the three most important predictors of lung involvement severity at the time of the first CT examination. Multivariable logistic regression analysis showed a significant strong association between the extent of the severity of lung involvement (continuous variable) and death; adjusted odds ratio (OR): 9.3; 95% CI: 7.1–12.1 in the TD and 2.6 (1.8–3.5) in the eVD.

**Conclusion:**

In hospitalized patients with COVID-19, the severity of lung involvement is a strong predictor of death. Age, CRP levels, and duration of hospitalizations are the most important predictors of severe lung involvement. A simple prediction model based on available clinical and imaging data provides a validated tool that predicts the severity of lung involvement and death probability among hospitalized patients with COVID-19.

## Introduction

Severe acute respiratory syndrome—coronavirus-2 (SARS-CoV2) has caused a fatal pandemic that has become an intense global health threat (Rothan and Byrareddy, 2020). Although most patients with severe coronavirus disease 2019 (COVID-19) may experience respiratory dysfunction, the disease can also cause severe complications in other organ systems, e.g., cardiovascular, neurological, and renal dysfunction contributing to death (Gavriatopoulou et al., 2020). The reported clinical manifestations of COVID-19, thus, far have indicated substantial heterogeneity in the prognosis of COVID-19 infection, spanning from asymptomatic patients to those with mild, moderate, and severe disease forms with low survival rates (Borges do Nascimento et al., 2020).

Several potential risk factors of poor prognosis in patients with COVID-19 have been reported. For instance, previous studies indicated that old age, male sex, and chronic comorbidities including hypertension, cardiovascular diseases (CVD), diabetes, and malignancies are associated with a higher mortality rate in critically ill patients with laboratory-confirmed COVID-19 (Parohan et al., 2020). Other predictors such as oxygen saturation (SaO_2_), immunosuppressive therapies, and neurological complications, including *de novo* seizure, headache, and delirium, are also related to poor outcomes in patients with COVID-19 (Louapre et al., 2020). Biomarkers on admission, such as D-dimer level, may also predict mortality risk (Ponti et al., 2020).

The diagnostic role of chest computed tomography (CT) in assessing the severity of COVID-19 infection has already been highlighted. Previous studies investigated predictors of COVID-19 exacerbation according to chest CT scans and showed factors such as serum albumin, C reactive protein (CRP), and coronary plaque burden as predictors of COVID-19 outcomes (Supplementary Table 1) (Arkoudis et al., 2022; Fukumoto et al., 2022; Inoue et al., 2022; Ke et al., 2022; Koch et al., 2022; Lu et al., 2022; Wang et al., 2022; Yang et al., 2022). However, these studies were limited by a low number of patients included, thus limited power to conclude. The lack of cross-validations also limited the findings' generalizability in previous studies.

Therefore, in the current study, we studied the predictive values of individuals' risk profiles in the extent of lung involvement according to chest CT scans in a large dataset that included hospitalized patients with COVID-19 infection in Iran. We further studied if lung lesion severity and its risk predictors could help predict the likelihood of mortality among these patients. The current study applied machine learning techniques to develop a prediction model in a training dataset (TD). The model was further validated in internal (iVD) and external (eVD) validation datasets.

## Methods

### Study setting and study population

**Figure 1:**
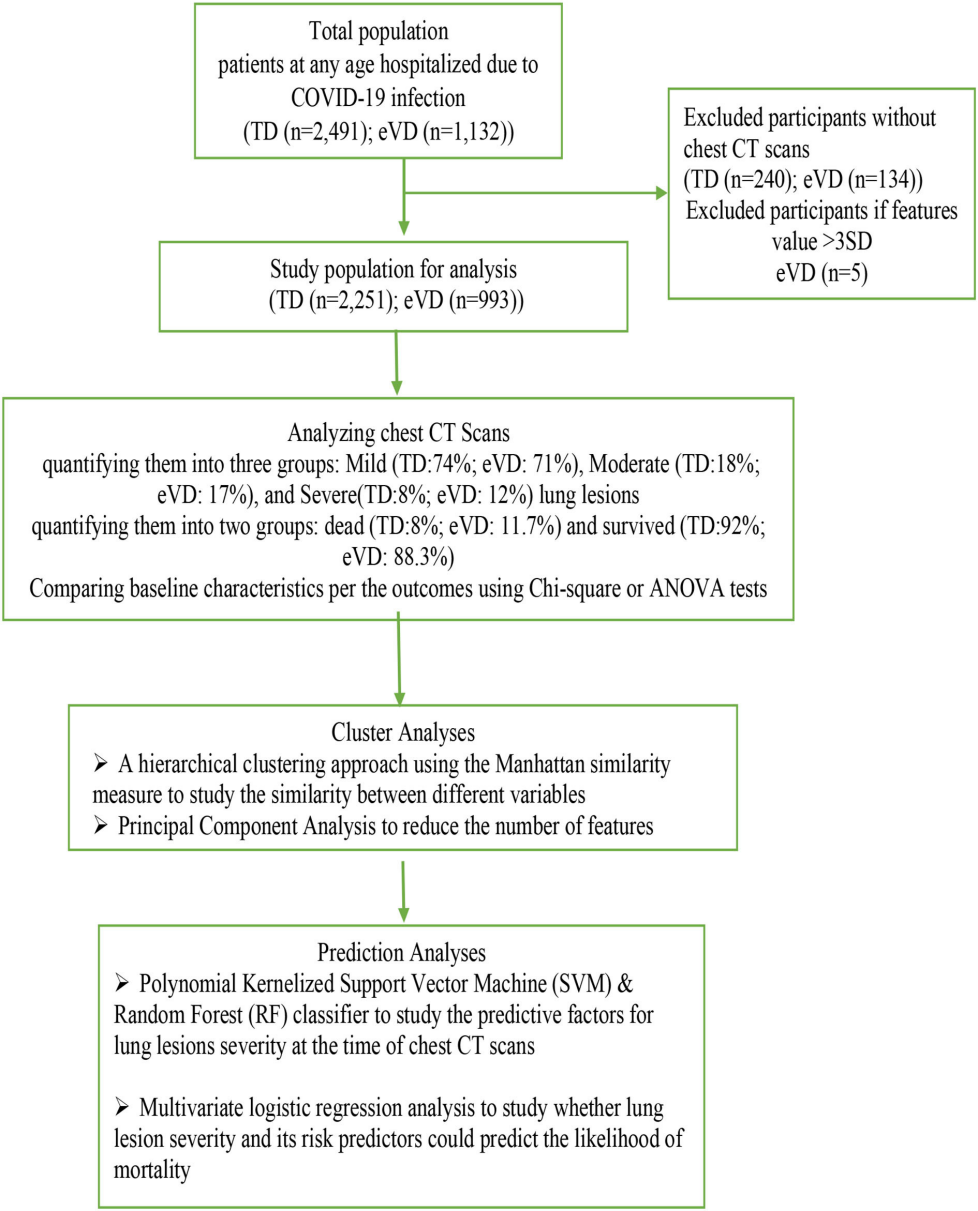
Flowchart of the study population. TD, training dataset; eVD, external validation dataset.

We used prospectively collected data on 2,491 (TD) and 1,132 (eVD) patients with positive polymerase chain reaction (PCR) hospitalized in Kurdistan University hospitals (Kavsar and Besat) in Sanandaj, a capital city of Kurdistan Province in the west of Iran. Data collected were from 20 February 2020 to 21 April 2021- the first, the second, and third waves of COVID-19 infection in Iran and before starting COVID-19 vaccination. After applying in and exclusion criteria, our datasets included 2,251 patients in the TD (Kavsar hospital) and 993 in the eVD (Besat hospital) for our analyses; patients of all ages with the CT data evaluating lung involvement were enrolled (Figure 1). None of the individuals included had received vaccination at the time of hospitalization. The Medical Ethics Committee of Kurdistan University of Medical Sciences has approved this study (registration number: IR.MUK.REC.1400.114).

### Assessment of predictors and ascertainment of outcomes

Data on twenty-five demographic and clinical symptoms and diseases (Supplementary Table 2) were collected at the CT examination through the hospital health information system (HIS). An internist (ER) and a pulmonologist (NS) reviewed the patients' medical records and abstracted this information.

According to the Regional Health Authorities Protocol and the patient's consent, a chest CT scan was performed in all PCR- positive patients with a SaO_2_ rate of <95%. The CT examination was done identically for all eligible patients during hospitalization. Non-contrast enhanced CT images were acquired on an 80-row scanner (Aquilion Prime S.P., Canon Medical Systems), with parameters based on the patient's morphotype (tension 100e135 kV and maximum mAs 2e50).

The primary endpoint was the severity of lung involvement expressed by the first CT scan scores performed during hospitalization. The day of first CT imaging was different for each patient and varied from 1 to 32 days in the TD and 1 to 37 days in the eVD after hospital admission (for each patient, the duration of hospitalization was calculated at the first CT examination). In each dataset, two radiologists performed visual quantification of the lung involvement in the CT; they were blinded without knowing the patients' clinical conditions. The consensus was made through consultation with a pulmonologist (NS) in case of discrepancies. We calculated a total CT scan score for each patient as the sum of the individual lobar scores that range from 0 (no involvement) to 25 (maximum involvement) when all the five lobes showed more than 75% involvement. The percentage of lung involvement affected by COVID-19 infection was calculated by multiplying the total score times four (Revel et al., 2020). The CT images were then classified as per the percentage of lung involvement in the following three groups applying the advice from the European Society of Radiology (ESR) and the European Society of Thoracic Imaging (ESTI) (Revel et al., 2020): mild (≤25%), moderate (26–50%), and severe >50% (Revel et al., 2020). The secondary endpoint was in-hospital death due to COVID-19 infection.

### Validation

We performed internal and external validation to study the accuracy of the fitted prediction models. In contrast to internal validation methods, which evaluate the model's performance in individuals from the same dataset/population, external validation evaluates model performance in new individuals from different but related practices that investigate the model's generalizability (Debray et al., 2015). We randomly split the TD into a training set (70%) and a test set (30%) for the internal validation.

### Statistical analysis

Baseline characteristics are presented as mean [standard deviation (*SD*)] for continuous variables and frequency (percentage) for categorical variables. In the complete case analyses, we compared each variable between different groups in the two datasets (the TD and the eVD) using the Chi-square (categorical variables) and ANOVA (continuous variables) statistical tests. To study predictors of the severity of lung involvement, we applied two supervised machine learning techniques, including (i) the Polynomial Kernelized Support Vector Machine (SVM) (Ben-Hur et al., 2001) and (ii) the random forest (RF) classifier (Breiman, 2001). We further compared the results to select the best technique for our analyses. According to the best technique (RF in our study), a prediction model was created. The complete prediction methods, statistics, and procedures are presented in the Supplementary Material. We also applied multivariable logistic regression analysis to study the association between severity of lung involvement and mortality risk presenting an adjusted odds ratio (OR) and 95% confidence interval (CI). The association was adjusted for age, sex, comorbidities, smoking, and respiratory distress.

The accuracy of the prediction models was evaluated using the area under the receiver operating characteristic (AUC-ROC) curves. A *p* < 0.05 was considered statistically significant. All analyses were performed using SPSS (version 26.0) (SPSS Inc., Chicago, IL, USA) and R (Mice version 3.14.0; Ggplot2 version 3.3.5; randomForest version 4.6-14; dendextend version 1.15.2) software.

## Results

### Baseline characteristics

According to the severity of lung involvement, patients were categorized into three groups: mild [1,663 (73.9%) and 708 (71.3%)], moderate [403 (17.9%) and 169 (17.0%)], and severe [185 (8.2%) and 116 (11.7%)] lung involvement, in the TD and the eVD, respectively. They were also categorized as dead [180 (8%) and 116 (11.7%)] and survived [2,071 (92%) and 877 (88.3%)] in the TD and the eVD, respectively; the rate of in-hospital death was substantially higher among patients with severe (64.9 and 38.8%) compared to mild (2.3 and 7.1%) and moderate (5.5 and 12.4%) lung involvement in the TD and eVD, respectively.

Comparisons of baseline clinical symptoms and comorbidities stratified by the outcomes of interest are presented in Table 1.

**Table 1:** Baseline characteristics of the patients with COVID-19 according to the extent of lung involvement and in-hospital death.

	**Training dataset (TD)**							**Validation dataset (VD)**						
	***n** =* **2,251**							***n** =* **993**						
	≤**25%** **(*****n** =* **1,663)**	**26-50%** **(*****n** =* **403)**	>**50%** **(*****n** =* **185)**	***P*** **-value**	**Survived** **(*****n** =* **2,071)**	**Dead** **(*****n** =* **180)**	****P*** **-value***	≤**25%** **(*****n** =* **708)**	**26–50%** **(*****n** =* **169)**	>**50%** **(*****n** =* **116)**	***P*** **-value**	**Survived** **(*****n** =* **877)**	**Dead** **(*****n** =* **116)**	****P*** **-value***
Male, *n* (%)	929 (55.9)	232 (57.6)	114 (61.6)	0.3	1,161 (56.1)	114 (63.3)	0.06	390 (55.1)	97 (57.4)	72 (62.1)	0.3	492 (56.1)	67 (57.8)	0.7
Female, *n* (%)	734 (44.1)	171 (42.4)	71 (38.4)		910 (43.9)	66 (36.7)		323 (45.6)	72 (42.6)	44 (37.9)		390 (44.5)	49 (42.2)	
Age, mean (*SD*), years	57.1 (17.9)	58.4 (18.4)	64.2 (16.9)	<0001	57.1 (18.0)	67.9 (16.0)	<0.001	52.1 (17.4)	51.2 (17.6)	56.2 (18.9)	0.04	51.1 (17.6)	62.6 (14.2)	<0.001
Previous COVID-19, *n* (%)	12 (0.7)	1 (0.2)	1 (0.5)	0.6	13 (0.6)	1 (0.6)	0.9	10 (1.4)	3 (1.8)	2 (1.7)	0.9	13 (1.5)	2 (1.7)	0.8
Smoking status (current), *n* (%)	18 (1.1)	2 (0.5)	1 (0.5)	0.5	20 (1)	1 (0.6)	0.6	38 (5.4)	8 (4.7)	7 (6.0)	0.9	45 (5.1)	8 (6.9)	0.4
^*^Comorbidities, *n* (%)	294 (17.7)	64 (15.9)	45 (24.3)	0.04	352 (17)	51 (28.3)	<0.001	157 (22.2)	31 (18.3)	24 (20.7)	0.6	183 (20.9)	29 (25)	0.3
Lung disorders, *n* (%)	24 (1.4)	8 (2.0)	5 (2.7)	0.4	18 (0.9)	8 (4.4)	<0.001	5 (0.7)	3 (1.8)	0	0.2	8 (0.9)	0	0.3
Duration of hospitalization, mean (*SD*), days	3.8 (3.2)	4.1 (3.7)	4.2 (4.7)	0.007	3.8 (3.2)	5.1 (5.5)	<0.001	4.3 (3.7)	4.7 (4.3)	5.4 (5.0)	0.008	4.1 (3.5)	7.1 (5.6)	<0.001
Clinical symptoms														
Fever, *n* (%)	559 (33.6)	147 (36.5)	62 (33.5)	0.6	710 (34.3)	58 (32.2)	0.6	323 (45.6)	64 (37.9)	45 (38.8)	0.1	383 (43.7)	49 (42.2)	0.8
Cough, *n* (%)	590 (35.5)	153 (38.0)	43 (23.2)	0.002	749 (36.2)	37 (20.6)	<0.001	307 (43.3)	76 (45.0)	45 (38.8)	0.6	380 (43.3)	48 (41.4)	0.7
^**^Respiratory distress, *n* (%)	698 (42.0)	168 (41.7)	47 (52.4)	0.02	861 (41.6)	102 (56.7)	<0.001	328 (46.3)	85 (50.0)	70 (60.3)	0.01	418 (47.7)	65 (56)	0.08
SaO_2_, <93%, *n* (%)	900 (54.1)	265 (65.8)	120 (64.9)	<0.001	1,164 (56.2)	121 (67.2)	0.004	377 (53.2)	93 (55.0)	86 (74.1)	<0.001	465 (53.0)	91 (78.4)	<0.001
Intubation rate, *n* (%)	76 (4.6)	18 (4.5)	35 (18.9)	<0.001	86 (4.2)	43 (23.9)	<0.001	40 (5.6)	9 (5.3)	16 (13.8)	0.003	54 (6.2)	11 (9.5)	0.2
Muscle pain, *n* (%)	782 (47.0)	176 (43.7)	73 (39.5)	0.09	969 (46.8)	62 (34.4)	0.001	351 (49.6)	178 (46.2)	58 (50.0)	0.7	430 (49.0)	57 (49.1)	0.9
Loss of consciousness, *n* (%)	71 (4.3)	12 (3.0)	26 (14.1)	<0.001	71 (3.4)	38 (21.1)	<0.001	41 (5.8)	9 (5.3)	16 (13.8)	0.004	59 (6.7)	7 (6)	0.7
Smell loss, *n* (%)	21 (1.3)	10 (2.5)	2 (1.1)	0.2	32 (1.5)	1 (0.6)	0.3	45 (6.4)	7 (4.1)	8 (6.9)	0.5	56 (6.4)	4 (3.4)	0.2
Taste loss, *n* (%)	23 (1.4)	6 (1.5)	5 (2.7)	0.4	30 (1.4)	4 (2.2)	0.4	32 (4.5)	7 (4.1)	7 (6.0)	0.7	39 (4.4)	7 (6)	0.4
Seizure, *n* (%)	4 (0.2)	1 (0.2)	0	0.8	5 (0.2)	0	0.5	0	0	1 (0.9)	0.02	0	1 (0.9)	0.006
Gastrointestinal disorders, *n* (%)	130 (7.8)	16 (4.0)	15 (8.1)	0.02	148 (7.1)	13 (7.2)	1.0	115 (16.2)	27 (16.0)	11 (9.5)	0.2	143 (16.3)	10 (8.6)	0.03
Nausea, *n* (%)	97 (5.8)	23 (5.7)	11 (5.9)	1.0	122 (5.9)	9 (5)	0.6	55 (7.8)	17 (10.1)	3 (2.6)	0.06	72 (8.2)	3 (2.6)	0.03
Vomiting, *n* (%)	63 (3.8)	11 (2.7)	7 (3.8)	0.6	73 (3.5)	8 (4.4)	0.5	33 (4.7)	8 (4.7)	5 (4.3)	1.0	43 (4.9)	3 (2.6)	0.3
diarrhea, *n* (%)	21 (1.3)	6 (1.5)	3 (1.6)	0.7	28 (1.4)	2 (1.1)	0.8	9 (1.3)	3 (1.8)	4 (3.4)	0.2	14 (1.6)	2 (1.7)	0.9
Anorexia, *n* (%)	39 (2.3)	9 (2.2)	6 (3.2)	0.7	52 (2.5)	2 (1.1)	0.3	27 (3.8)	10 (5.9)	6 (5.2)	0.4	37 (4.2)	6 (5.2)	0.6
Headache, *n* (%)	106 (6.4)	40 (9.9)	12 (6.5)	0.04	149 (7.2)	9 (5)	0.3	73 (10.3)	24 (14.2)	10 (8.6)	0.2	92 (10.5)	15 (12.9)	0.4
Dizziness, *n* (%)	99 (6.0)	37 (9.2)	14 (7.6)	0.05	141 (6.8)	9 (5)	0.4	50 (7.1)	17 (10.1)	5 (4.3)	0.2	65 (7.4)	7 (6)	0.6
Paresthesia, *n* (%)	36 (2.2)	8 (2.0)	3 (1.6)	0.9	44 (2.1)	3 (1.7)	0.7	20 (2.8)	6 (3.6)	2 (1.7)	0.7	23 (2.6)	5 (4.3)	0.3
Plegia, *n* (%)	9 (0.5)	4 (1.0)	3 (1.6)	0.2	14 (0.7)	2 (1.1)	0.5	2 (0.3)	1 (0.6)	0	0.7	1 (0.1)	2 (1.7)	0.003
Chest pain, *n* (%)	7 (0.4)	4 (1.0)	2 (1.1)	0.3	11 (0.5)	2 (1.1)	0.3	14 (1.8)	6 (3.6)	1 (0.9)	0.3	18 (2)	3 (2.6)	0.7
Laboratory findings														
CRP, mg/L, mean (*SD*),	7.1 (14.6)	10.9 (13.6)	17.8 (15.4)	<0.001	12.4 (14.5)	17.4 (14.5)	0.04	7.9 (10.3)	8.7 (11.6)	13.6 (15.2)	<0.001	6.9 (9)	21.8 (16.8)	<0.001
Outcomes														
Death, *n* (%)	38 (2.3)	22 (5.5)	120 (64.9)	<0.001	-	-	-	50 (7.1)	21 (12.4)	45 (38.8)	<0.001	-	-	-

*SD, standard deviation; CRP, C reactive protein; SaO_2_, oxygen saturation*.

* *Comorbidities were defined as a composite of skin disorders, cancer, liver disorders, diabetes, blood disorders, immune disorders, CVD, renal disorders, or psychological disorders*.

** *Respiratory distress was defined as a respiratory rate higher than 24*.

*Bold values are statistically significant p-values (p value < 0.05)*.

In the TD, the mean (*SD*) age was 57.1 (17.9) years in patients with mild, 58.4 (18.4) years in moderate, and 64.2 (16.9) years in severe lung involvement. Patients with severe lung involvement were significantly older than the other two groups (*p* < 0001). In the eVD, patients were, on average, 8 years younger than those in the TD; the mean (SD) age was 52.1 (17.4) years in patients with mild, 51.2 (17.6) years in moderate, and 56.2 (18.9) years in severe lung involvement. Patients with severe lung involvement were significantly older than the other two groups (*p* = 0.04).

In the two datasets, the distribution of men was higher than women across different groups, with the highest among patients with severe lung involvement and a higher rate of in-hospital death.

The mean (*SD*) of the duration of hospitalization (days) was higher in severe lung involvement [4.2 (4.7) in the TD and 5.4 (5.0) in the eVD] than in groups with mild and moderate lung involvement.

In the TD, cough, respiratory distress, loss of consciousness, gastrointestinal disorders, headache, and dizziness at first CT examination were among clinical symptoms with statistically significant differences between patients in different categories of lung involvement severity. Prevalence of comorbidities such as diabetes and CVD was significantly higher in patients with severe lung involvement (24.3%) than mild (17.7%) and moderate (15.9%) lung involvement. The SaO_2_ value was significantly lower among patients with moderate and severe than mild lung involvement. The intubation rate was significantly higher among patients with severe lung lesions (18.9%), whereas the rates were lower and almost similar in mild (4.6%) and moderate (4.5%).

In the eVD, respiratory distress, SaO_2_ values and intubation rate, loss of consciousness, and seizure were statistically higher among patients with severe lung involvement than those categorized with mild or moderate involvement.

In both datasets, C reactive protein (CRP) levels were significantly higher among patients with severe lung involvement (Table 1).

The results of cluster analyses in both TD and eVD are presented in Supplementary Materials (Supplementary Figures 1, 2).

### Prediction analysis

#### Analyses of the training dataset

##### Random forest

**Figure 2:**
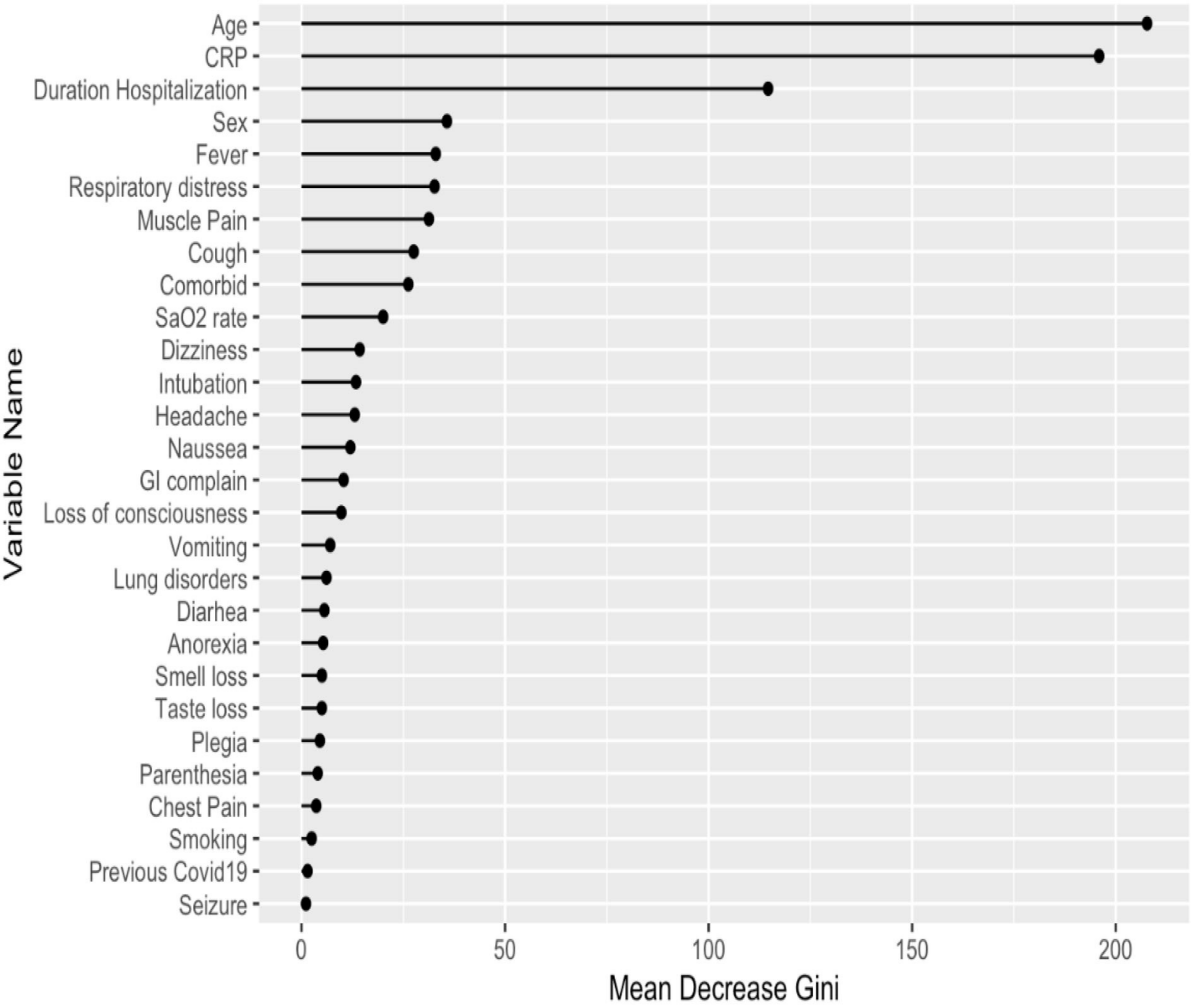
Predictors of lung involvement severity in hospitalized patients with COVID-19 infection in the training dataset. CRP, C reactive protein; GI, gastrointestinal.

Our study is composed mostly of categorical variables. Despite having a heterogeneous distribution of outcomes (most of our population had mild lung involvement or were alive), the RF showed a higher predictive power than the SVM method; therefore, the RF has been selected as the best technique for our analyses. Theoretically, our dataset has a good fit for a decision tree since the decisions made by the random forest would be easily understood if a subject had or did not have a certain, e.g., clinical symptom. The RF analysis and the Mean Decrease Accuracy plot showed age, CRP levels, and duration of hospitalizations with the highest predictive value for lung involvement (Figure 2).

#### Analyses of the validation dataset

The analyses in both internal and external VDs provided similar results to the TD for the RF algorithms (Supplementary Figure 3). The confusion matrices showed similar precision and sensitivity during the testing phase.

Logistic regression analysis showed a significant strong association between the extent of lung involvement (as a continuous variable) and mortality risk in the adjusted model [(OR: 9.3; 95%CI:7.1–12.1) and (OR: 2.6; 95%CI:1.8–3.5)] in the TD and the eVD, respectively and with a high discrimination value of AUC-ROC >0.8 in both datasets.

**Figure 3:**
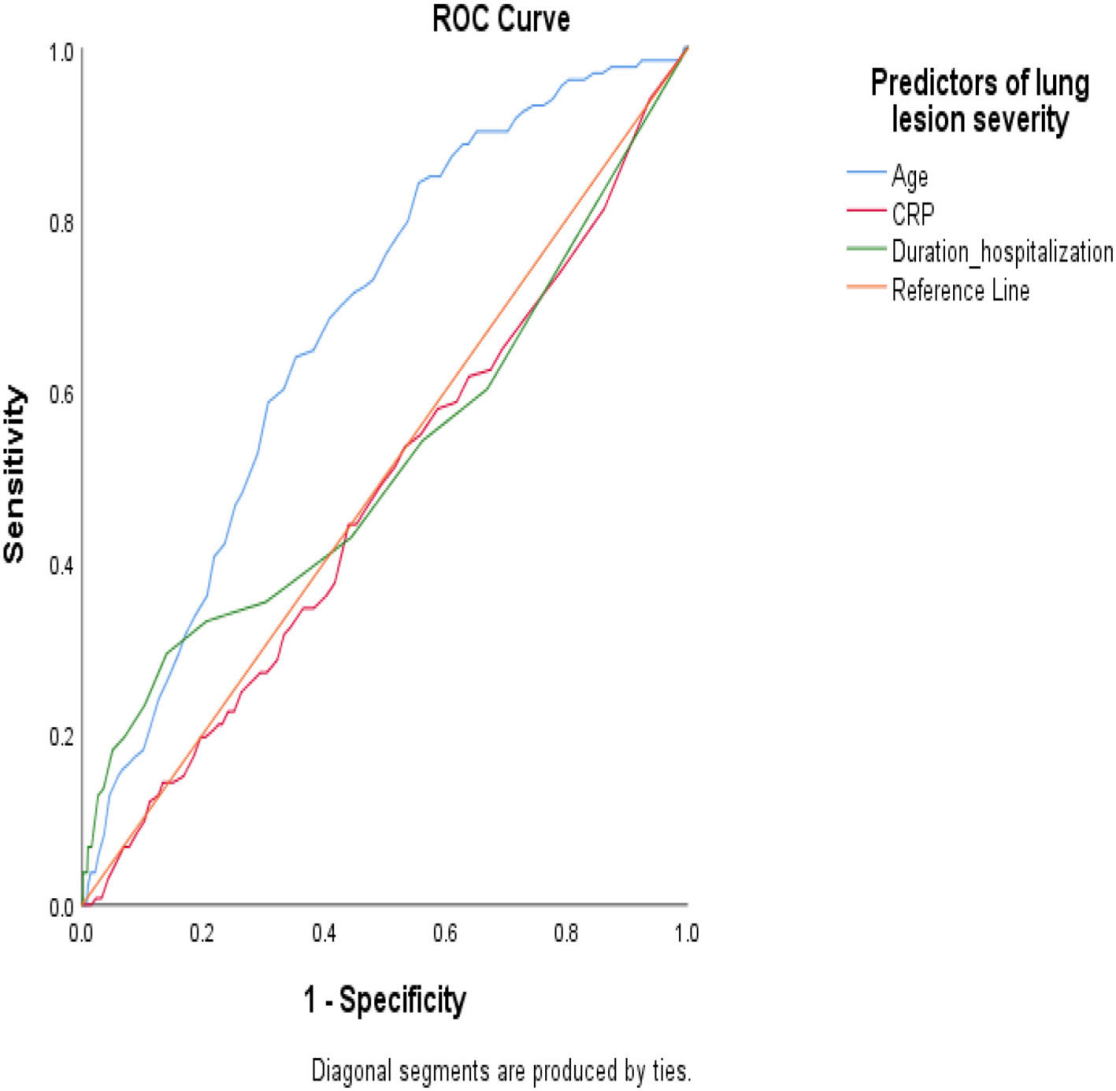
Under the receiver operating characteristic (AUC-ROC) curve to study the predictive value of the most significant predictors of lung involvement severity in the likelihood of death in hospitalized patients with COVID-19 infection (training dataset).

To study whether predictors of lung involvement are associated with mortality in this population, we studied the AUC-ROC values. The results showed that predictors of lung involvement failed to discriminate between patients who died compared to survivors in the TD (AUC-ROC <0.7) (Figure 3). However, the results showed a high discrimination value of CRP levels (AUC-ROC >0.8) in the eVD (Supplementary Figure 4).

## Discussion

We developed and validated a prediction model applying machine learning techniques that showed good performance and discrimination in training and validated datasets. To our best knowledge, this is the largest study to support the values of patients' symptoms and risk profiles to predict the severity of lung involvement and in-hospital death due to COVID-19 infection. Our study showed that in hospitalized patients with COVID-19 disease, age, CRP level, and duration of hospitalizations were factors in patients' risk profiles that can discriminate the extent of lung involvement. However, these factors failed to predict death (AUC-ROC <0.7) except CRP in the eVD. In the group of severe lung involvement, patients were older, and the prevalence of comorbidities, respiratory distress, intubation rate, and loss of consciousness was significantly higher than in mild and moderate lung involvement. Our findings suggest a strong prognosis value of the extent of lung involvement to predict mortality with high discrimination power (AUC-ROC >0.8); the majority of patients who died in the hospital due to COVID-19 had severe lung involvement (65% in the TD).

The initial COVID-19 strain caused serious disease, and the virus will cohabit with humans, probably forever. The number of infected people with COVID-19 and complications due to infection is rapidly growing. It is important to prioritize patients with severe COVID-19 for intensive care unit (ICU) admission, particularly in low and middle-income countries with limited resources and high demand (Tyrrell et al., 2021). There is an urge to identify early biomarkers of COVID-19 severity that may help effective treatment strategies. COVID-19 disease is mainly characterized by symptoms such as fever, weakness, muscle pain, cough, headache, and high levels of CRP and inflammatory cytokines, with severe symptoms involving multiple organs; respiratory failure, acute cardiac, and kidney injuries result in patients' death eventually. The scientific community should develop strategies to fight the illness by developing preventive strategies and effective therapies. At the same time, it is necessary to develop means to mitigate the effects of any unusual circumstances, such as during the surge in cases with severe COVID-19 (Kim et al., 2021; Van Hout and Wells, 2021). For COVID-19, predictive modeling is used broadly to help in different purposes. For example, predicting fatal outcomes in hospitalized patients based on symptom severity enables physicians to prioritize patients for ICU admission and mechanical ventilation and relieve such decisions' ethical burdens and concerns (Wynants et al., 2020; Merlo et al., 2021).

We observed that the top predictors of lung involvement severity in hospitalized patients with COVID-19 are age, CRP level, and duration of hospitalization. Similarly, previous evidence indicated that the incidence of fatalities from a COVID-19 outbreak depends crucially on the infected age groups. This is hazardous not only for elderlies but also for middle-aged adults (Levin et al., 2020). A meta-analysis with more than half a million patients with COVID-19 from different countries highlights the determinant effect of age on mortality (Bonanad et al., 2020). In addition, Wang et al. found that CT findings of multi-affected lobes were more commonly seen in elderly patients than in young patients (Wang et al., 2020). Older adult patients should be prioritized in implementing preventive measures (Sudharsanan et al., 2020). Elevated CRP level indicates the severity of COVID-19 in many cases, and it has been introduced as the main predictor for mechanical ventilation in patients with COVID-19 (Zhang et al., 2020) and reflects inflammatory reaction (Yitbarek et al., 2021). Various inflammatory mediators induce CRP produced by hepatocytes in the liver, e.g., interleukin (IL)-6, and it is associated with chronic inflammations (Luan and Yao, 2018). In our study, patients with COVID-19 and severe lung involvement were in the hospital longer than the other two groups. Patients who experience severe symptoms of COVID-19 may spend weeks to months in the hospitals (Liu et al., 2020). Their need for more care and therapies might explain this, and one might view longer duration as a consequence of infection severity. However, this needs to be assessed to rule out reverse causality in which longer hospitalization may lead to complicated lung involvement due to pneumonia and other concomitant hospital infections. Long time duration of hospitalization is associated with the risk of secondary infection such as hospital-acquired pneumonia, which can affect the survival rate of COVID-19 infected people (Langford et al., 2020).

Despite we developed a prediction model and identified predictors of lung involvement, these predictors failed to discriminate between patients who died compared to survivors in the TD. However, the predictive value of CRP in mortality rate was shown in the eVD. Unlike our result in the TD, a study in the United Kingdom evidenced CRP as one of the most accurate predictors of death (Stringer et al., 2021); this might be explained by the low rate of mortality due to the short time of death investigation (in-hospital mortality), and single CRP measurements in our study. Hence, future studies need to evaluate this.

Previous prediction studies highlighted the history of comorbidities as one of the important factors associated with mortality risk in patients infected with COVID-19 (Lopez-Pais et al., 2021; Oh et al., 2021; Ryan et al., 2021). Though the rate of comorbidities, respiratory distress, and intubation was statistically higher in patients with severe lung involvement, our models failed to pick these factors as strong predictors of COVID-19 outcomes. Besides, we showed that patients with severe lung involvement than mild and moderate had a higher loss of consciousness, which is interpreted as decreased SaO_2_ level; loss of consciousness is associated with cerebral hypoxia and other neurological complications due to COVID-19 infections (Bentivegna et al., 2021; Kannapadi et al., 2022).

While our study is not the first prediction model of COVID-19-related outcomes, to the best of our knowledge, this is the largest prediction study involving CT scan data. Our model strengths also include the validation in a large external dataset. This study effectively assists clinical decision-making for combating patients with COVID-19 by providing the risk of severe lung involvement and death. Our study helps clinicians pragmatically prioritize high-risk hospitalized patients according to their risk profile to prevent severe outcomes related to COVID-19 infection. However, caution is warranted when generalizing these findings to other populations and settings; the results may not be generalized to populations with different geographical and socioeconomic conditions and national health services. Another important limitation of our study that should be acknowledged is the retrospective nature of this study; we were not able to follow the extent of lung lesions and the association with mortality rate after discharging. We believe future studies should investigate how severe lung involvement changes after discharge in hospitalized patients with COVID-19. Patients in our datasets were enrolled during the three pandemic waves in Iran; therefore, the prediction model may not fit during other epidemic periods with different virus strains and the severity of the infection.

## Access to data

ER and FA have access to the data. This data is accessible after a motivated request.

## Data availability statement

The raw data supporting the conclusions of this article will be made available by the authors, without undue reservation.

## Ethics statement

The studies involving human participants were reviewed and approved by the Medical Ethics Committee of Kurdistan University of Medical Sciences. The patients/participants provided their written informed consent to participate in this study.

## Author contributions

ER and FA are responsible for the study concept and design. ER and MA collected the data. FA, AR, and SEB composed the statistical analyses. ER, MSh, and FA wrote the manuscript. FA is the guarantor of this study and takes responsibility for the integrity and accuracy of the data analysis. All the authors contributed to interpreting the data, critical revision of the manuscript, and approved the submitted version.

## Conflict of interest

The authors declare that they have no known competing financial interests or personal relationships that could have appeared to influence the work reported in this article.

## Publisher's note

All claims expressed in this article are solely those of the authors and do not necessarily represent those of their affiliated organizations, or those of the publisher, the editors and the reviewers. Any product that may be evaluated in this article, or claim that may be made by its manufacturer, is not guaranteed or endorsed by the publisher.
