# Supplementary material for: The risk profile of patients with COVID-19 as predictors of lung lesions severity and mortality—Development and validation of a prediction model
Source: Front Microbiol. 2022 Jul 25;13:893750. doi: 10.3389/fmicb.2022.893750 (PMC9361066; doi:10.3389/fmicb.2022.893750)
Supplement: Supplementary file 1 [file Data_Sheet_1.PDF]

Supplementary materials

Table S1. Summary of previous studies investigating predictors of COVID-19 related outcomes

Study	Title	Outcomes	Methods	Predictors	Limitations
Inoue A,2022 (7)	Application of the advanced lung cancer inflammation index for patients with coronavirus disease 2019 pneumonia: Combined risk prediction model with advanced lung cancer inflammation index, computed tomography and chest radiograph	COVID-19 exacerbation	Applying random forest classification to combine Advanced lung cancer inflammation index (ALI) and radiologic risk prediction model.	ALI and chest CT score	This study was designed retrospectively with a limited number of patients. Moreover, the survival rate was not determined and no external validation.
Koch V,2022 (8)	Lung Opacity and Coronary Artery Calcium Score: A Combined Tool for Risk Stratification and Outcome Prediction in COVID-19 Patients.	Pulmonary involvement in patients with COVID-19 patients with pneumonia	Pulmonary involvement was evaluated with a dedicated software prototype by two experienced radiologists and expressed as Opacity Score.	Coronary plaque burden	This study was designed retrospectively with a limited number of patients. The laboratory parameters were collected only at the peak of symptoms without considering dynamic changes over time, and no external validation.
Fukumoto W,2022 (9)	Triaging of COVID-19 patients using low dose chest CT: Incidence and factor analysis of lung involvement on CT images.	Incidence of lung involvement	Multivariate logistic regression with cross-validation on clinico-laboratory variables	Patient age, albumin, lactate dehydrogenase, and C-reactive protein	This study was designed retrospectively with a limited number of patients. The interval between the acquisition of PCR-positive results and CT scans varied among patients. In addition, the cut-off value of clinico-laboratory data used may have slightly affected the ROC curve of the prediction model.

Lu X,2022 (10)	The association of obesity with the progression and outcome of COVID-19: The insight from an artificial-intelligence-based imaging quantitative analysis on computed tomography.	severe pneumonia lesions on CT	Applying the clinical-plus-AI parameter model	Obesity	This was a single-center study, and the sample size was limited. In addition, other serum risk factors such as D-dimer, IL-6, and troponin were not available in most of the patients, thus not included in the risk prediction models, and no external validation.
Arkoudis NA,2022 (11)	Covid Visual Assessment Scale ("Co.V.A.Sc."): quantification of COVID-19 disease extent on admission chest computed tomography (CT) in the prediction of clinical outcome-a retrospective analysis of 273 patients.	ICU admission and in-hospital death	The possible correlation between clinical and laboratory variables with ICU admission or death was evaluated using binary regression analysis.	Covid Visual Assessment Scale ("Co.V.A.Sc.") for chest CT (the total extent of pulmonary parenchyma affected by COVID-19-associated pulmonary opacities), patient age, gender	This was a retrospective, single-center study, and the sample size was limited, and no external validation.
Ke Z,2022 (12)	Radiomics analysis enables fatal outcome prediction for hospitalized patients with coronavirus disease 2019 (COVID-19).	Fatal outcome of COVID-19 pneumonia	A radiomics signature was generated using the least absolute shrinkage and selection operator (LASSO) Cox regression model.	Age, temperature, pulse oxygen saturation, initial oxygen therapy mode, and lymphocyte count	This study was designed retrospectively with a limited number of patients.
Yang B,2022 (13)	Heterogeneity Analysis of Chest CT Predict Individual Prognosis of COVID-19 Patients.	Death and the prolonged median length of hospital stay in COVID-19 patients	Applying histogram heterogeneity analysis based on CT opacities.	Ground-glass Opacity (GGO) and Consolidation Opacity (CLO)	Lack of longitudinal study conduction with only analyzing the onset of COVID-19 pneumonia. Second, they did not include asymptotic patients in this retrospective study, and no external validation.
Wang R,2022 (14)	Artificial intelligence for prediction of COVID-19 progression using CT imaging and clinical data.	ICU admission and mechanical ventilation and death during a	Development of an artificial intelligence (AI) system in a time-to-event analysis framework	Age, presence of fever, elevated white blood cell count, Lymphocyte count, preexisted comorbidities	There was likely patient selection bias secondary to the retrospective and

		hospital stay in patients with COVID-19			multi-institutional nature of the study.

Table S2. List of potential predictors of the severity of lung involvement

**Predictors**	**Continuous variable**	**Categorical variable (yes/no)**
Age	X	
Sex (male and female)		X
Fever (temperature above 37 °C)		X
Cough		X
Muscle pain		X
Chest pain		X
Respiratory distress (a respiratory rate higher than 24)		X
Consciousness loss		X
Smell loss		X
Taste loss		X
Sizure		X
Gastrointestinal disorder		X
Nausea		X
Intubation		X
Oxygen saturation (Po2 rate <93%)		X
Headache		X
Diarrhea		X
Dizzness		X
Paraesthesia		X
Anorexia		X
Smoking		X
Lung disorder/asthma		X
Comorbidities defined as a composite of skin disorders, cancer, liver disorders, diabetes, blood disorders, immune disorders, CVD, renal disorders, psychological disorders, chronic disorders, or hypertension		X
C reactive protein (CRP)	X	
Duration of hospitalization	X	

Methods/Statistical analysis

For the prediction analyses, a hierarchical clustering approach using the Manhattan similarity measure was first used to analyze the similarity between the different variables. We also performed a Principal Component Analysis (PCA) to cluster the predictors and reduce the number of features. In these clustering approaches, we included 25 demographic and clinical symptoms and diseases listed in **Table S2**. We excluded outliers (features values >3SD), and all continuous variables were log-transformed and scaled to having 0 means and SD as one.

For the prediction analysis, we applied two supervised machine learning techniques, including (i) the Polynomial Kernelized Support Vector Machine (SVM) and (ii) the random forest (RF) classifier, and

created a predictive model according to the best model. The Polynomial Kernelized SVM method is good at dealing with high-dimensional spaces trained using a (0.75,0.25) testing set ratio and a 10-fold cross-validation methodology. This method was trained using all the features from the dataset (K=2). The confusion matrix of the results using the extent of lung involvement and death as classes presents the number of correct and incorrect predictions with count values for each class. The first 15 principal components (PCs) were used to train a kernelized SVM according to the cumulative summation curve of the Proportion of Variance (Pov).

The RF classifier is based on decision trees which split the data at decision nodes according to feature values. RF is composed of a combination of decision trees (bagging) where each forest is trained from a random selection of features and a random partition of training and testing datasets. We can determine which variables contribute more to the decrease per predictor within the trees, which presents the percentage of accuracy lost if a specific predictor is excluded. The Mean Decrease Gini plot expresses the importance of each variable within the RF; the higher the percentage, the more important it. The RF was applied with CT scan scores and death as outcomes.

Results

Cluster analysis

The proportion of variance of a PCA for the TD is presented in **Table S3**. Using the Manhattan similarity measure, a hierarchical clustering approach presented two very distinct groups shown by the cluster dendrogram (**Figure S1**). Sex, age, fever, cough, respiratory distress, SaO_2 values, muscle pain, duration of hospitalization, and CRP levels were clustered in a group different from the other cluster.

Table S3: The proportion of variance of a Principal Component Analysis

	**PC1**	**PC2**	**PC3**	**PC4**	**PC5**	**PC6**	**PC7**	**PC8**
**Standard deviation**	1.0387	1.0001	0.9838	0.54452	0.5232	0.50173	0.49580	0.44734

**Proportion of Variance**	0.2113	0.1959	0.1895	0.05806	0.0536	0.04929	0.04813	0.03918
**Cumulative Proportion**	0.2113	0.4071	0.5966	0.65469	0.7083	0.75757	0.80571	0.84489

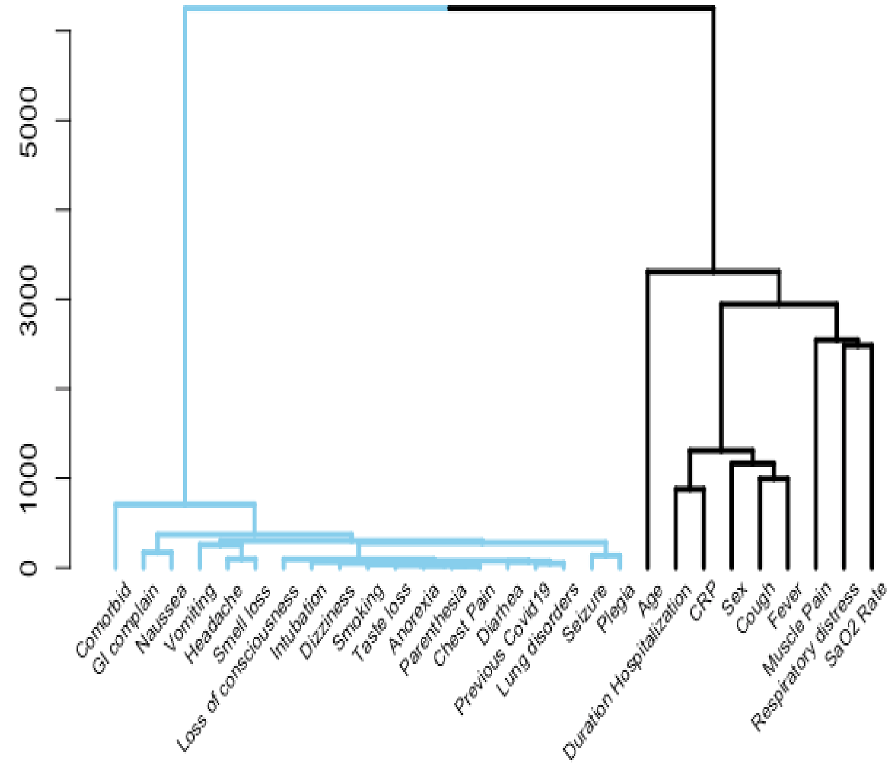

Figure S1: Cluster dendrogram to study similarity between potential predictors in the training dataset

Abbreviation: CRP, C reactive protein

The results in the external validation dataset are presented below (**Figure S2**).

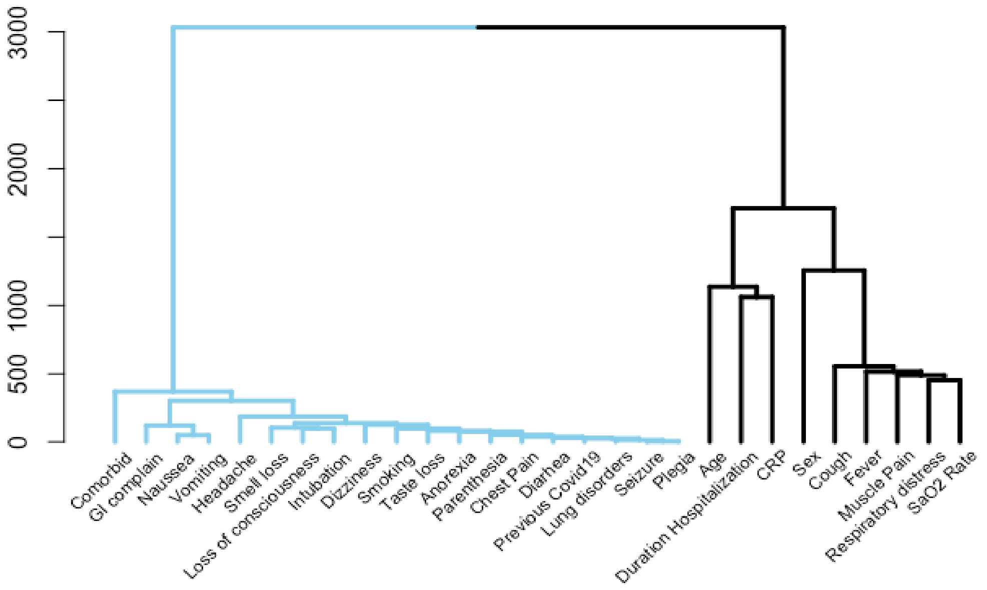

Figure S2. Cluster dendrogram to study similarity between potential predictors (external validation dataset)
Abbreviation: CRP, C reactive protein

Prediction analysis

Polynomial Kernelized Support Vector Machine. The results of the Polynomial Kernelized SVM present a confusion matrix of the results using the CT scan scores and death as classes. The confusion matrix presents the number of correct and incorrect predictions with count values for each class. Our results showed no differences between using features/predictors separately and clusters to predict the outcomes (data not shown).

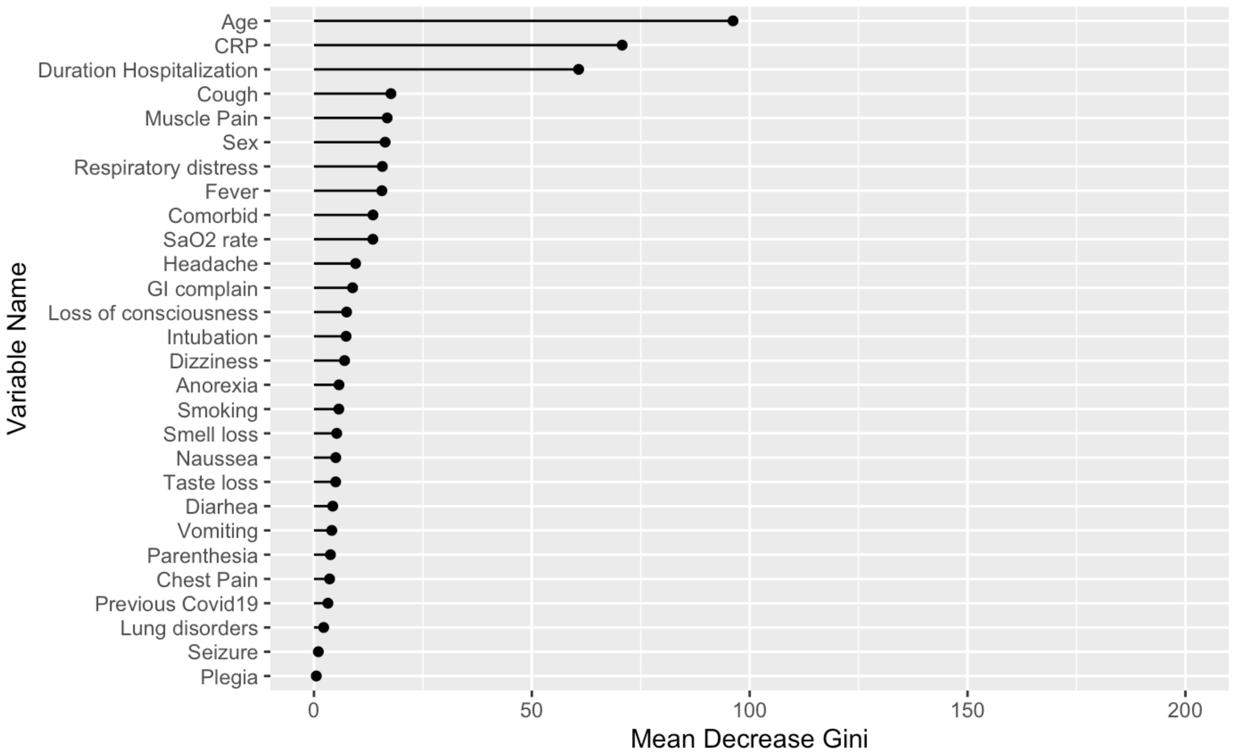

Figure S3: Predictors of lung lesions severity in hospitalized patients with COVID-19 infection (external validation dataset)

Abbreviations: CRP, C reactive protein; GI, gastrointestinal

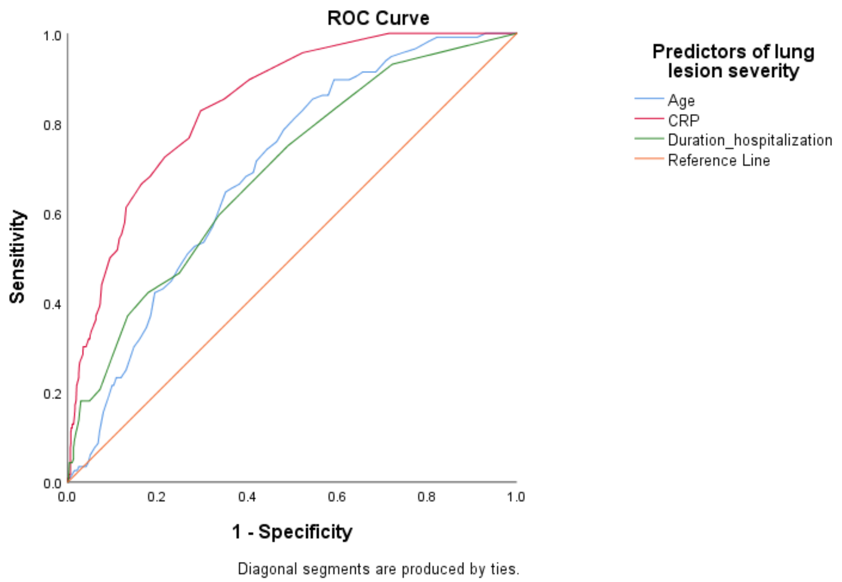

Figure S4. Under the receiver operating characteristic (AUC-ROC) curve to study the predictive value of the most significant predictors of lung involvement severity in the likelihood of death in hospitalized patients with Covid-19 infection (external validation dataset)

**Factors**	**AUC (95% CI)**
Age	0.69 (0.65-0.74)
CRP (C reactive protein)	0.84 (0.81-0.88)
Duration of hospitalization	0.69 (0.64-0.74)
